# Rapid Profiling of Metabolites Combined with Network Pharmacology to Explore the Potential Mechanism of *Sanguisorba officinalis* L. against Thrombocytopenia

**DOI:** 10.3390/metabo12111074

**Published:** 2022-11-05

**Authors:** Yubei Dai, Kailian Zhang, Long Wang, Ling Xiong, Feihong Huang, Qianqian Huang, Jianming Wu, Jing Zeng

**Affiliations:** 1 School of Pharmacy, Southwest Medical University, Luzhou 646000, China; 2 School of Basic Medical Science, Southwest Medical University, Luzhou 646000, China; 3 Education Ministry Key Laboratory of Medical Electrophysiology, Southwest Medical University, Luzhou 646000, China; 4 Key Medical Laboratory of New Drug Discovery and Druggability Evaluation, Southwest Medical University, Luzhou 646000, China; 5 Key Laboratory of Activity Screening and Druggability Evaluation for Chinese Materia Medica, Southwest Medical University, Luzhou 646000, China

**Keywords:** herbal medicine, *Sanguisorba officinalis* L., metabolites, thrombocytopenia, network pharmacology

## Abstract

*Sanguisorba officinalis* L. (*SO*), a well-known herbal medicine, has been proven to show effect against thrombocytopenia. However, metabolites of *SO* in vivo are still unclear, and the underlying mechanism of *SO* against thrombocytopenia from the aspect of metabolites have not been well elucidated. In this study, an improved analytical method combined with UHPLC-QTOF MS and a molecular network was developed for the rapid characterization of metabolites in vivo based on fragmentation patterns. Then, network pharmacology (NP) was used to elucidate the potential mechanism of *SO* against thrombocytopenia. As a result, a total of 1678 exogenous metabolites were detected in urine, feces, plasma, and bone marrow, in which 104 metabolites were tentatively characterized. These characterized metabolites that originated from plasma, urine, and feces were then imported to the NP analysis. The results showed that the metabolites from plasma, urine, and feces could be responsible for the pharmacological activity against thrombocytopenia by regulating the PI3K-Akt, MAPK, JAK-STAT, VEGF, chemokine, actin cytoskeleton, HIF-1, and pluripotency of stem cells. This study provides a rapid method for metabolite characterization and a new perspective of underlying mechanism study from the aspect of active metabolites in vivo.

## 1. Introduction

Herbal medicine (HM) plays a vital role in modern biological science and demonstrates definite curative effects; however, its mechanism of action requires further study. The metabolic analysis of HM in vivo is a systematic task aimed at the qualitative and quantitative analysis of metabolites in the living body [1], which is significantly helpful for understanding potential mechanisms.

Modern analytical technologies including nuclear magnetic resonance (NMR), high–performance liquid chromatography (HPLC), gas chromatography (GC), capillary electrophoresis (CE) and their corresponding tandem mass spectrometry methods (LC–MS, GC–MS, and CE–MS) [2,3,4,5,6] have been developed for metabolic analysis. Ultrahigh-performance liquid chromatography (UHPLC) tandem high-resolution MS is an excellent technique that provides high separation efficiency, high sensitivity, high mass accuracy, and structurally related information [7,8,9]. The metabolites of the Tao-Hong-Si-Wu decoction [10], Gui-Zhi-Jia-Ge-Gen decoction [11], *Achyranthes* [12], Xian-Ling-Gu-Bao capsule [13], Ge-Gen-Qin-Lian pill [14], *Phellodendri amurensis* cortex and Zhibai Dihuang pill [15], Wu-Tou Decoction [4], Deng-Zhan-Xi-Xin injection [16], and *Angelicae Pubescentis* Radix [17] have been successfully characterized by UHPLC tandem quadrupole time-of-flight MS (UHPLC-QTOF MS). Moreover, Fourier transform ion cyclotron resonance MS [18], the recently developed linear ion trap-Orbitrap mass spectrometry [19], and Q-Exactive Orbitrap tandem MS [20] were successively employed for metabolic profiling with an extremely high resolution, demonstrating a significantly enhanced metabolite characterization. Considering the difficulties of the metabolite data profiling of HMs, a target integration strategy [21], “representative structure-based homologous xenobiotics identification” (RSBHXI) strategy [13,22], manual MS network [23], “four-step” integrated strategy [24], and “five-step” strategy [1] were developed to improve the depth of profiling and to simplify metabolite characterization and pathway analysis. However, the comprehensive metabolite profiling of HM in vivo is still challenging, owing to its extremely complex composition and tedious data analysis.

As far as we know, metabolites are structurally similar compounds of prototype ingredients that share the same product ions or neutral loss ions in a collision-induced dissociation. Huan et al. developed a core structure-based search algorithm based on a hypothetical neutral loss for spectral similarity analysis and an unknown metabolite annotation [25]. Hsu et al. developed a strategy based on multiple types of correlated ion information for metabolite identification [26]. To facilitate data analysis, Bandeira et al. developed a molecular network (MN) which was a visualization platform for the analysis of untargeted MS/MS data and it was also a web-based MS data ecosystem [27]. Based on structurally similar compounds or analogs that produce similar fragmentation patterns, MN automatically compares and clusters the spectra of each precursor ion, and the resulting networks are plotted. Compounds from different samples can be marked using different colors, and the relative content of each metabolite can be shown using a pie chart. The compounds are clustered into different families in MN, facilitating the simultaneous characterization of abundant and trace compounds. This method has been successfully employed to analyze antibiotics [28], peptides [29], marine organisms [30,31], and components of HMs [32]. However, the metabolic analysis of HMs using the multifunctional MN has not been reported.

Network pharmacology (NP) was first proposed by Hopkins [33]. Owing to the viewpoint of “multi-constituents, multi-targets, and synergistic effects” of HM, NP has been widely used to prediction the molecular mechanisms of HMs [34]. Many NP studies have focused on the reported compounds from various databases to explore pharmacological mechanisms. However, few studies have been based on the metabolites in vivo, which have been proven to be the potential active constituents [35]. In NP, the network of predicted associations among drugs, targets, pathways, and disease for a particular group of biological samples by experimental results or database information can be summarized and visualized. For example, the connected nodes represent diverse biological entities which including drugs, genes, pathways, and disease. The edges represent the predicted functional associations, such as coexpression evidence among genes. Relying on this clustered network with high-throughput technologies, the potential relationship between drugs and diseases can be predicted.

In this study, a rapid metabolite profiling method was established for the characterization and visualization of metabolites of *Sanguisorba officinalis* L. (*SO*) in vivo, which are commonly used as a HM against thrombocytopenia (TCP) [36]. Profiled metabolites in plasma, urine, and feces were subsequently imported to perform an NP analysis to explain the molecular mechanism of *SO* against TCP. As a result, 1678 metabolites (repeated metabolites were not included) were detected, of which 913, 601, 364, and 160 metabolites were detected in the urine, feces, plasma, and bone marrow, respectively. The ingredients of *SO* in mice were mainly excreted through urine and feces. A total of 104 metabolites with a cosine value >0.7 were tentatively characterized. The NP analysis revealed that metabolites from plasma, urine, and feces could be responsible for the pharmacological activity of thrombocytopenia by regulating the following pathways in cancer: PI3K-Akt, MAPK, JAK-STAT, VEGF, chemokine, actin cytoskeleton, HIF-1, and pluripotency of stem cells.

## 2. Experimental Methods

### 2.1. Chemicals and Materials

Acetonitrile and formic acid were of LC–MS grade (Thermo Fisher, MA, USA). A water purification system (Milli-Q, Billerica, MA, USA) was used to generate ultrapure water. *SO* was obtained from Chengdu Jinhong Co., Ltd. (Chengdu, China) and confirmed using its morphological characteristics. All other reagents were of analytical grade.

### 2.2. Preparation of SO Extract

*SO* (100 g) was soaked in 800 mL of ultrapure water for 30 min and extracted with water at 100 °C thrice. The extracts were combined and concentrated using a rotary evaporator for intragastric administration.

### 2.3. Animal Experiments

Here, 12 mature Sprague Dawley (SD) rats (6 males and 6 females) weighing 30 ± 3 g (mean ± standard deviation) were provided by Southwest Medical University (Luzhou, Sichuan, China) and used. The rats were maintained within a temperature range of 20–25 °C and in a 12 h light/dark cycle. Prior to the drug administration, the rats were acclimated to the environment for seven days with free access to food and water. The rats were randomly divided into two groups (*n* = 6 per group, comprising three males and three females), i.e., the normal control group and the *SO* group (250 mg/kg, treatment). Thereafter, the rats were placed on a fast with free drinking water during the test. Plasma, urine, and fecal samples were collected 0.5, 1, and 2 h after oral administration. Immediately after the biosample collection, the rats were sacrificed, and the thighbones were stripped out to collect their bone marrows. All procedures were reviewed and approved by the Biomedical Ethical Committee of Southwest Medical University (no. 20211123-014) and were consistent with animal care guidelines.

### 2.4. Sample Preparation

Plasma samples (200 μL) were obtained by centrifugation at 3500 rpm (4 °C) for 15 min, and 600 μL of acetonitrile was added to 200 μL of plasma. The mixture was vortexed for 3 min, let stand for 10 min, and centrifuged at 10,000 rpm for 15 min at 4 °C. The supernatant was transferred to a sample vial for detection.

The urine samples were immediately stored at −70 °C until analysis. They were thawed on ice prior to injection and similarly processed as the plasma samples.

Freeze-dried feces (80 mg) were mixed with 800 μL of 80% methanol/water. The mixture was ultrasonicated in an ice bath for 30 min, and centrifuged at 10,000 rpm for 15 min at 4 °C. The supernatant (200 μL) was collected for further analyses.

The bone marrow was flushed using a 1.5 mL saline rinse via a syringe into a centrifuge tube, and the collected sample was added to methanol (600 μL). The sample was ultrasonicated in an ice bath for 30 min and centrifuged at 10,000 rpm (4 °C) for 15 min. The supernatant was collected for further analysis.

### 2.5. Chromatographic and MS Conditions

The MS/MS data were obtained using a UHPLC (Exion)-QTOF (X500R) MS system (Sciex, MA, USA). Chromatographic separation was performed using an Exion UHPLC system (Sciex, MA, USA) coupled to a Kinetex C_18_ column (100 × 2.1 mm, 2.6 μm, 100 Å) (Phenomenex, CA, USA) at 40 °C. Mobile phase A was 0.1% formic acid/water (*v*:*v*), while mobile phase B was acetonitrile. The flow rate was set at 0.2 mL/min. The gradient elution was 5% B from 0 to 2.00 min, 5–50% B from 2.01 to 18.00 min, and 50–100% A from 18.01 to 20.00 min. The injection volume was 20 μL.

An X500R QTOF MS system was equipped with a Turbo V^TM^ source and a Twin Sprayer probe for electrospray ionization (ESI) (Sciex, MA, USA). The source parameters for positive and negative polarity were as follows: temperature, 500 °C; full scan mass range, *m/z* 100–1500; ion source gases 1 and 2, 50 psi; curtain gas, 35 psi; and collisionally activated dissociation (CAD) gas, 7 psi.

The automated MS/MS data acquisition was performed in the information-dependent acquisition (IDA) mode. The specific parameters with dynamic background subtraction were as follows: maximum candidate ions, 10; intensity threshold exceedances, 200 cps; and former candidate ions were excluded for 5 s after 1 occurrence.

### 2.6. Metabolite Data Acquisition and Preprocessing

The SCIEX OS 1.4 software (Sciex, MA, USA) was used to acquire and output the raw data files, which were converted into mzXML format using the MSConvert software. The transformed files were uploaded to the MN platform (http://gnps.ucsd.edu (accessed on 1 April 2022)) using the FileZilla software. Moreover, the minimum pair cosine score was set to 0.7, the minimum cluster size was set to 2, and the minimum matched fragment ions were set to 6 before MN was created. Furthermore, endogenous metabolites were eliminated by subtracting the data of normal control group. These parameters could be manually modified before generating the MN. The default values were used for the remaining parameters, and the MN was visualized using Cytoscape 3.7.1 (NIGMS, MD, USA).

### 2.7. Network Pharmacology Analysis

Swisstarget Prediction was used to obtain and download the prediction targets of the plasma, urine, and feces metabolites of *SO*. With “thrombocytopenia” as the keyword and limiting the species to “Homo sapiens”, the disease targets were obtained by using GeneCards databases (https://www.genecards.org (accessed on 29 April 2022)), DisGeNET (https://www.disgenet.org/search (accessed on 29 April 2022)), Drugbank (https://www.drugbank.ca/ (accessed on 29 April 2022)), Online Mendelian Inheritance in Man (https://www.omim.org (accessed on 29 April 2022)), PharmGKB (https://www.pharmgkb.org (accessed on 29 April 2022)), and Therapeutic Target Database (http://db.idrblab.net/ttd/ (accessed on 29 April 2022)); duplicate targets were removed.

The *SO* and TCP-related targets were input into the STRING database (http://string-db.org/ (accessed on 1 June 2022)) to predict the possible protein–protein interaction (PPI) information. Then, the PPI data with degree values ≥28, ≥47, and ≥58 of plasma, urine, and feces were reserved. By using Metascape (http://metascape.org/gp/index.html#/main/step1 (accessed on 1 June 2022)), the Kyoto Encyclopedia of Genes and Genomes (KEGG) pathway and gene ontology (GO) enrichment evaluations were performed to clarify the pathways that were involved in metabolites acting on TCP.

## 3. Results and Discussion

Here, a rapid metabolite analysis method was developed, which included four steps: (1) the acquisition of MS/MS data by UHPLC-QTOF MS, (2) construction of a MN, (3) identification of metabolites originating from different tissues by library searching and MN-guided annotation, (4) visualization of origins and relative quantification of metabolites in different tissues using pie charts in different colors, and (5) summarization of metabolic pathways. Thereafter, these metabolites from different tissues were used for the NP analysis.

### 3.1. Optimization of LC–MS Conditions

To characterize metabolites with different properties, generality should be adequately considered at the beginning of method development. A long-standing C_18_ column and the general gradient used in this study started from 5% and ended at 100% acetonitrile within 20 min. This maintained the polar component in the column as much as possible and eluted the nonpolar component from the column during the running period since baseline resolution was not necessary for the MS/MS data acquisition and processing. ESI, which is regarded as the most popular soft ionization source, was used in this method because it was significantly less affected by the matrix effect [37]. *SO*, blank biosamples (plasma, urine, and feces), and rat’s plasma, urine, and feces after administration of *SO* were analyzed and compared. As shown in Figure S1, the constituent of *SO* is extremely complex, and the chromatograms between blank biosamples and biosamples after administration are quite different. The mass spectrometer was programed to perform a full scan within *m/z* 100–1500 in positive and negative ion detection modes. For the nontargeted MS data acquisition, the IDA mode was fully competent to record practically all the MS/MS spectra, although the IDA strategy, i.e., sequential window acquisition of all theoretical fragment ions (SWATH), emerged as an unbiased acquisition method. The high-resolution mass analyzer ensured that the qualitative analysis was accurate. The UHPLC/QTOF/MS platform combined with the UHPLC and MS conditions is a generally analytical strategy that facilitates the rapid analysis of various metabolites.

### 3.2. Construction of the Molecular Network of SO

**Figure 1:**
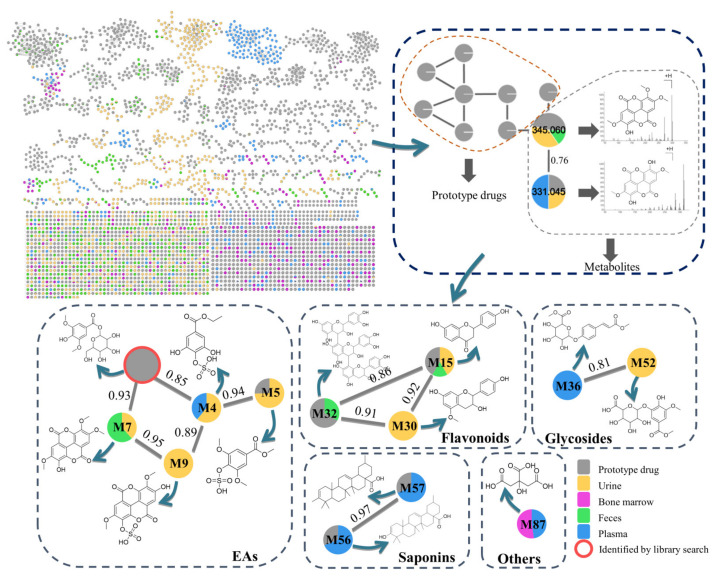
Metabolites molecular networks of *SO*.

In a MN, the connected nodes with a cosine value represent compounds with similar structures and fragmentation patterns. The closer the cosine value gets to one, the higher the structural similarity. In addition, the metabolites from different origins can be distinguished using different colors. As shown in Figure 1, green, yellow, blue, purple, and gray nodes represent their origins, including feces, urine, plasma, bone marrow, and the prototype ingredients of *SO*, respectively. Therefore, the absorption and distribution (plasma, digestive system, and urine) of these metabolites can be easily understood using different colors. Based on this established network, the absorption, distribution, metabolism, and excretion of *SO* was intuitively demonstrated. The yellow and green nodes originating from the urine and feces, respectively, were intuitively observed to be the dominant nodes and were statistically calculated as 41.9% and 35.5% of the 1678 nodes. A few metabolites were observed in the plasma (blue nodes) and bone marrow (purple nodes). Therefore, urinary metabolism is the main excretion pathway of *SO*, followed by fecal metabolism. Furthermore, the relative abundance of each metabolite from different origins was intuitively demonstrated using pie charts. For example, the relative content of **M7**, a metabolite of ellagic acids (EAs), as shown in Figure 1, was 40 and 60% in the urine and feces pathways, respectively. In addition, **M7** and **M9** exhibited a cosine value of 0.95, demonstrating a high structural similarity to each other. The substituents of **M9** were similar to that of **M7**, except for the occurrence of the sulfonic acidification of **M9**. **M7** was detected in urine and feces, while **M9** was only detected in urine.

### 3.3. Characterization of Metabolites

Previous studies have provided MS-based strategies [35,38,39] for metabolite profiling, combining a series of MS libraries [40] based on nontargeted metabolite profiling data. However, their applications have been limited by the number of spectra stored in libraries. Based on the method of a previous study [32], the MN established in this study clustered the structurally similar compounds into a network, greatly reducing the difficulties of characterization. In a network, a node represents a compound, and spectrum-to-spectrum alignments represent the edges between nodes. The connected nodes indicate compounds with similar fragmentation patterns and chemical structures. The obtained MS/MS spectra were searched against GNPS spectral libraries, in which the matched and unmatched compounds were illustrated. Ingredients undergo various metabolic transformations, such as hydroxylation, acetylation, and glucuronidation. The metabolic derivatives are a series of structurally similar compounds that possess the same molecular skeleton, resulting in similar fragmentation patterns during collision-induced dissociation. These similar fragmentation patterns can be recognized, clustered, and visualized using the MN, simultaneously facilitating the characterization of abundant and trace metabolites.

Here, 1678 metabolites of *SO* were detected, in which 104 metabolites were tentatively characterized and are detailed in Table S1. For all cases, once one compound was annotated in a cluster or matched by the libraries, the structure of the related nodes could be annotated from this node according to the similarity of fragment ions/neutral loss and accurate mass differences between nodes. Two metabolites with a cosine value greater than 0.7 were characterized.

#### 3.3.1. Feces

**Figure 2:**
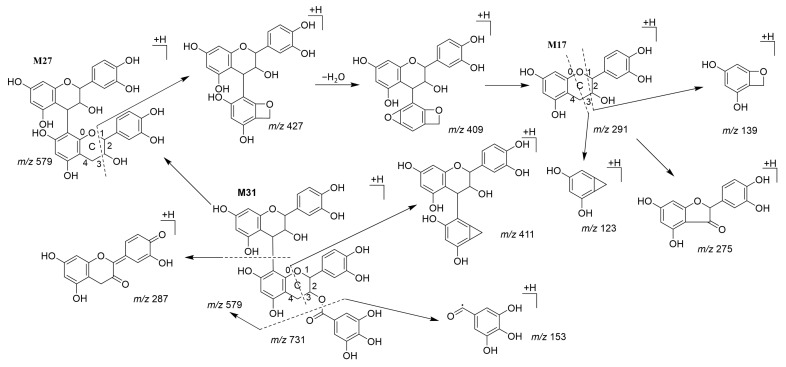
Fragmentation pathways of **M17**, **M27**, and **M31**.

Fifty prototype components and 16 metabolites of *SO* from the feces were characterized; the main components were flavonoids and saponins (Table S1). **M17** was detected in the feces and identified as catechin by a library search. As shown in Figure 2, **M17** exhibited fragmentation ions at *m/z* 139.0387 and 123.0438, corresponding to [M − ^1,3^B + H]^+^ and [M − ^0,3^B + H]^+^, respectively, which were undoubtedly the specific fragmentations of the C-ring opening of flavonoids [41]. Similar fragmentation pathways were observed in **M27** and **M31**. **M27** exhibited fragment ions at *m/z* 427.1038, 409.0925, and 275.0556, corresponding to the consecutive elimination of neutral molecular [M − ^1,3^B + H]^+^, [M − ^1,4^B + H]^+^, and [M − catechin + H]^+^, respectively. **M31** exhibited fragmentation ions at *m/z* 579.1129 [M + H − galloyl]^+^, 411.1066 [M + H − galloyl − ^1,4^B]^+^, and 153.0179 [gallic acid + H]^+^. Thus, they were identified as procyanidin B2 (**M27**) and procyanidin B2 3’-*O*-gallate (**M31**) [42]. Other flavonoid metabolites such as **M19**, **M20**, **M21**, and **M26** originated from the parent drug 4′,7-dihydroxyflavone conjugated with methyl, hydroxymethyl, and glycosyl under the action of intestinal microflora and were excreted in the feces. Other metabolic pathways including methylation, decarboxylation, glycosylation, and glucuronidation, which are common in intestinal metabolism [13,38,39,43], were detected in saponin metabolites, such as **M58**, **M60**, **M83**, and **M85**. Owing to the MS/MS collision, the **M83** presence of fragmentation ions at *m/z* 797.4654, 599.3913, and 441.3704 demonstrated a continuous neutral loss of rhamnose, glucose, and glucuronic acid. Furthermore, EAs were converted via lactone ring-opening, dihydroxylation, decarboxylation, and further glucuronidation into urolithin B-*O*-glucuronide (**M8**), which is the main metabolic pathway of EA derivatives in the gastrointestinal tract [44].

#### 3.3.2. Urine

Here, 19 parent drugs and 15 metabolites of *SO* were profiled in urine samples; the main components were EAs and glycosides (Table S1). EA metabolites in urine such as **M6**, **M10**, and **M11** are sulfated, methylated, and glucuronide forms of EA, respectively. Methyl EA and EA glucuronide are common metabolites from urine metabolism [45]. Similarly, the lactone rings of EAs were hydrolyzed into urolithin through urinary excretion and underwent decarboxylation and conjugation with methyl groups (**M2** and **M3**) [44,46]. Thus, the same neutral loss ions M–80 Da, which is common in natural products and represents the elimination of SO_3,_ were present in both **M4** and **M5**. Furthermore, EA prototype ingredients and metabolites were practically present in urine, demonstrating that the pathway through the urinary system may be the main metabolic pathway for EAs. The urinary glycoside metabolites were mostly *O*-glycosides generated from the dehydration of gallic acid and monosaccharides, followed by conjugation with methyl, sulfonic acid, and glucuronic acid groups.

#### 3.3.3. Plasma

Four prototype ingredients and six metabolites of *SO* from the plasma were characterized. The collision-induced fragmentation pathway of **M4** exhibited continuous neutral losses of the sulfonic and ethyl groups with specific fragments observed at *m/z* 197.0457 and 166.9989, respectively. **M36** exhibited fragment ion peaks at *m/z* 307.0830 and 277.0724, indicating the continuous neutral loss of the acetoxy and carbinol groups, respectively.

#### 3.3.4. Bone Marrow

There have been few studies on the metabolite analysis of *SO* in the bone marrow, although active ingredients isolated from *SO*, such as ziyuglycosides I and II have been demonstrated to improve hematopoiesis in vitro [47]. Here, four metabolites (**M87**, **M95**, **M98**, and **M99**) of *SO* from the bone marrow were characterized.

### 3.4. Analysis of Metabolic Pathways

The UHPLC combined MN allowed for the rapid elucidation of the possible metabolic pathways of *SO* in vivo. As shown in Table S1, the exogenous metabolites were tentatively characterized. As shown in Figures S2–S5, the metabolites were classified into four classes (EAs, flavonoids, glycosides, and saponins) based on different skeletons.

The metabolites belonging to these four classes with the same metabolic pathways were identified as methylation (**M5**-**7**, **M24**, **M29**, **M48**, **M49**, **M66**, and **M80**); glycosylation (**M11**, **M26**, **M29**, **M75**, **M78**, and **M81**); and glucuronidation (**M13**, **M22-24**, **M46**, **M64**, **M83**, and **M85**). The metabolites of EAs (**M4**, **M5**, **M9**, and **M10**) and glycosides (**M38** and **M41**) exhibited the same fragment ions M–80 Da, which demonstrated the metabolic pathway of sulfonation. Lactone opening pathways were observed in EAs, such as **M2**, **M3**, and **M8**. Flavonoids (**M31** and **M33**) and glycosides (**M38**, **M41**, and **M49**) with gallic acid conjugates were observed. Furthermore, dehydration and oxidation metabolites were present in the flavonoids (**M16**), glycosides (**M42** and **M51**), and saponins (**M58**, **M60**, **M63**, and **M67**).

### 3.5. Network Pharmacology Analysis

**Figure 3:**
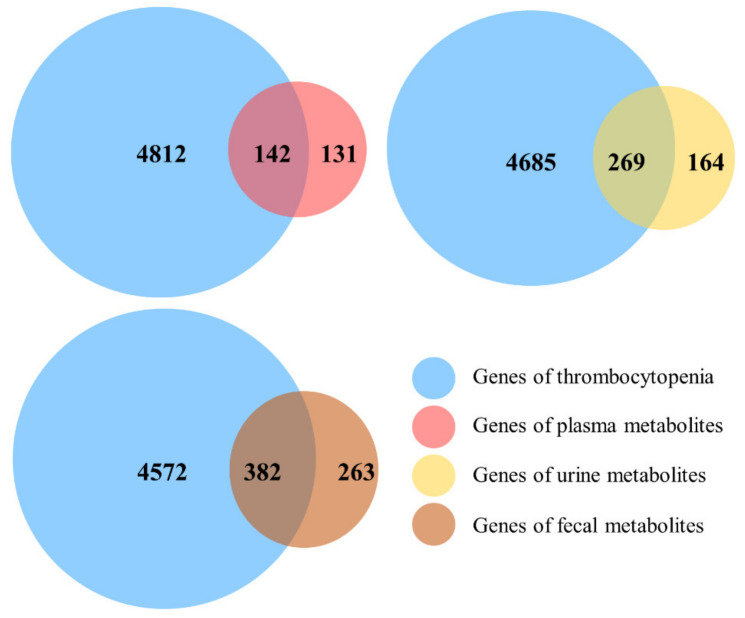
The Venn diagram of targets between thrombocytopenia and metabolites. The targets of thrombocytopenia were marked in blue, and the targets of metabolites from plasma, urine, and feces were marked in red, yellow, and brown, respectively.

Totally, 273, 433, and 645 targets were revealed corresponding to the plasma, urine, and feces metabolites, respectively. The target genes related to TCP were searched and 142, 269, and 382 common targets among metabolites from plasma, urine and feces and disease were identified, respectively (shown in Figure 3). Furthermore, the statistical results showed that 49%, 73%, and 81% common targets were different among the plasma, urine, and feces metabolites. This also confirms the multi-component and multi-target characteristics of HM.

**Figure 4:**
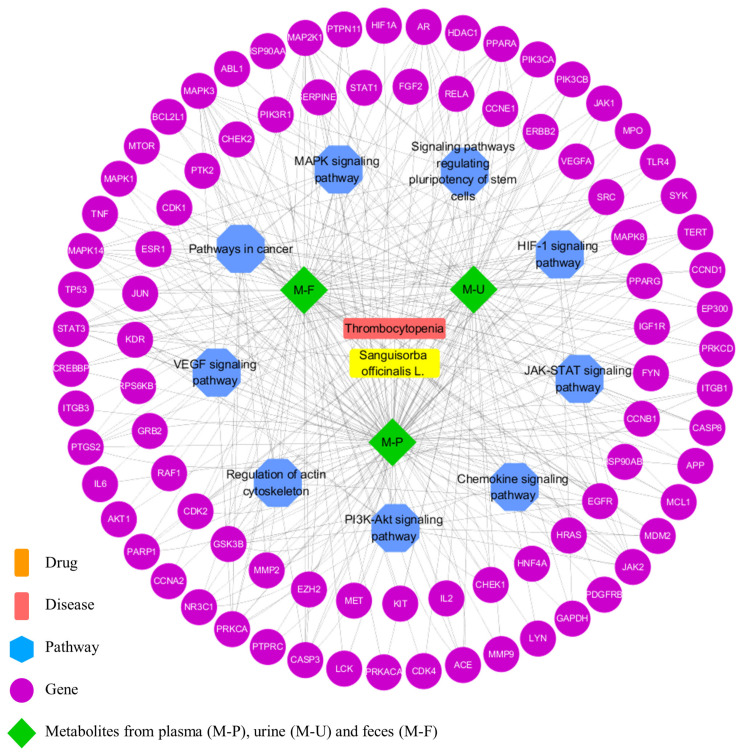
Targets/pathways/disease network of *SO* metabolites and thrombocytopenia. The targets of metabolites were marked in purple, the pathways were marked in blue, metabolites were marked in green, diseases were marked in red, and *SO* was marked in orange.

Meanwhile, the PPI network was constructed by using common targets, which were used to construct the metabolites-target-pathway network by using the Cytoscape software (shown in Figure 4). In the PPI network, the node with a higher score may be more important in the central correlation. According to the screening principle of degree value being larger than or equal to twice their respective median, 26, 57 and 82 key potential targets of plasma, urine, and feces metabolites with disease were screened out.

**Figure 5:**
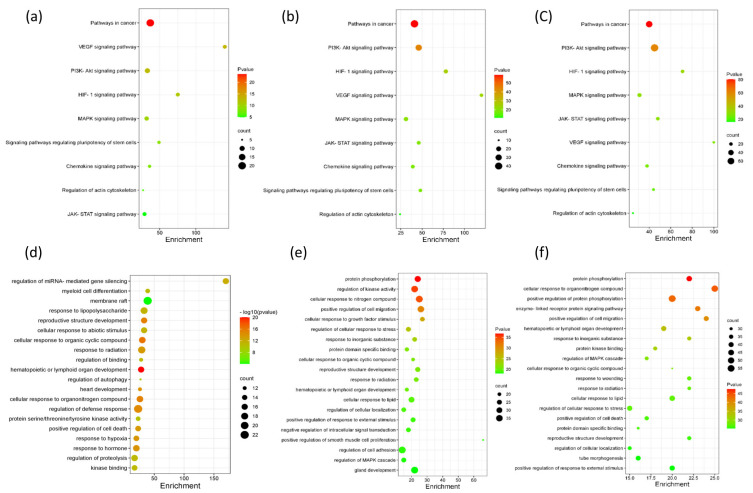
KEGG analysis (**a**–**c**) and Gene Ontology (**d**–**f**) enrichment analysis of the target proteins of metabolites. Metabolites from plasma (**a**,**d**), urine (**b**,**e**) and feces (**c**,**f**).

The KEGG database was utilized to note related pathways. A total of 168 pathways were obtained from key potential targets; nine pathways were probably the vital pathways, which possessed the count of significantly enriched (*p* value < 0.0001), comprising pathways in cancer, i.e., PI3K-Akt, MAPK, JAK-STAT, VEGF, chemokine, actin cytoskeleton, HIF-1, and pluripotency of stem cells (shown in Figure 5).

**Figure 6:**
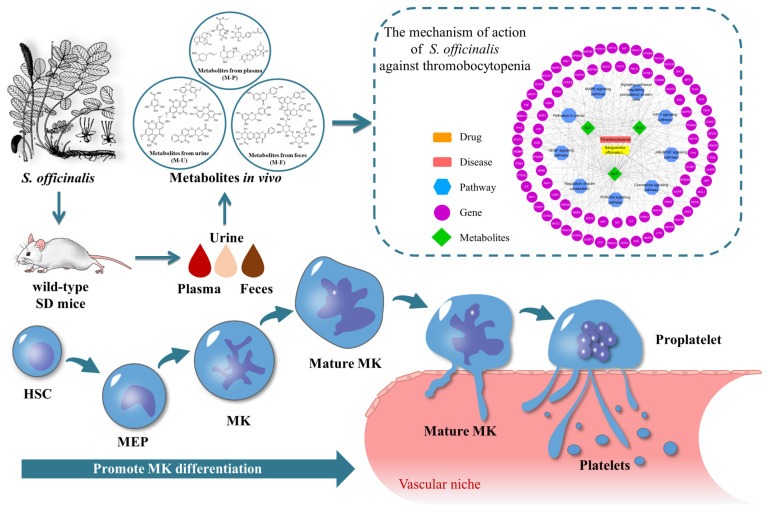
The underlying mechanism of *SO* against thrombocytopenia.

These enriched nine vital pathways were mainly associated with the hemopoietic system. PI3K-Akt was confirmed as a critical signaling pathway, which inhibits the aggregation and activation of platelets after allogeneic hematopoietic stem cell transplantation [48]. MAPK plays a vital role in the megakaryopoiesis differentiation process [49]. There was a study that demonstrated targeting JAK-STAT could lead to limiting treatment-emergent side effects, such as anemia and TCP [50]. In addition, the expression of HIF1A proteins in hematopoietic cells have been reported to cause lethal hemophagocytic lymphohistiocytosis-like phenotypes: severe anemia, TCP, splenomegaly, and multi-organ failure upon HIF1A induction [51]. An experimental study has demonstrated that VEGF-A was crucial for the hematopoietic stem and progenitor cell response during the early phase of acute TCP [52]. Hao et al. [53] showed that increased chemokine in plasma, such as CXC, was implicated in the pathogenesis of immune TCP. The relationship between regulation of actin cytoskeleton signaling pathway and TCP has also been summarized [54]. Therefore, as shown in Figure 6, the functions involved in the above signal pathways are inferred to be closely related to the promotion of MK differentiation, which requires further verification.

## 4. Conclusions

In this study, a rapid metabolite analysis strategy was developed for the comprehensive metabolite profiling of *SO* in vivo. Metabolites in plasma, urine, and feces were then used for NP analysis to deduce the potential mechanism. Ultimately, a total of 1678 metabolites were detected, of which 913, 601, 364, and 160 were present in the urine, feces, plasma, and bone marrow, respectively. In addition, 104 metabolites were tentatively identified, including 13 EAs, 20 flavonoids, 20 glycosides, 32 saponins, and 19 other compounds. The metabolites of *SO* in mice were mainly excreted through the urine and feces. The metabolic pathways of methylation, glycosylation, glucuronidation, sulfonation, lactone opening, gallic acid conjugates, dehydration, and oxidation were identified. Based on these characterized metabolites in vivo, the NP analysis reveals that the mechanism of *SO* against TCP is related to the following pathways in cancer: PI3K-Akt, MAPK, JAK-STAT, VEGF, chemokine, actin cytoskeleton, HIF-1, and pluripotency of stem cells.

## Data Availability

Data is contained within the article or supplementary material.

## Supplementary Materials

The following supporting information can be downloaded at: https://www.mdpi.com/article/10.3390/metabo12111074/s1, Figure S1: Total ion chromatograms of: (A) urine; (B) plasma; (C) feces, before and after administration of SO. (D) SO extract alone; Figure S2: Metabolites of ellagic acids (EAs) in vivo; Figure S3: Metabolites of flavonoids in vivo; Figure S4: Metabolites of glycosides in vivo; Figure S5: Metabolites of saponins in vivo; Table S1: Structures of the tentatively characterized compounds in *Sanguisorba officinalis* L.
