# Supplementary material for: Rapid Profiling of Metabolites Combined with Network Pharmacology to Explore the Potential Mechanism of Sanguisorba officinalis L. against Thrombocytopenia
Source: Metabolites. 2022 Nov 5;12(11):1074. doi: 10.3390/metabo12111074 (PMC9693491; doi:10.3390/metabo12111074)
Supplement: Supplementary file 1 [file metabolites-12-01074-s001.zip › metabolites-2008717-supplementary.pdf]

Supporting Information

Rapid Profiling of Metabolites Combined with Network Pharmacology to Explore the Potential Mechanism of *Sanguisorba officinalis* L. against Thrombocytopenia

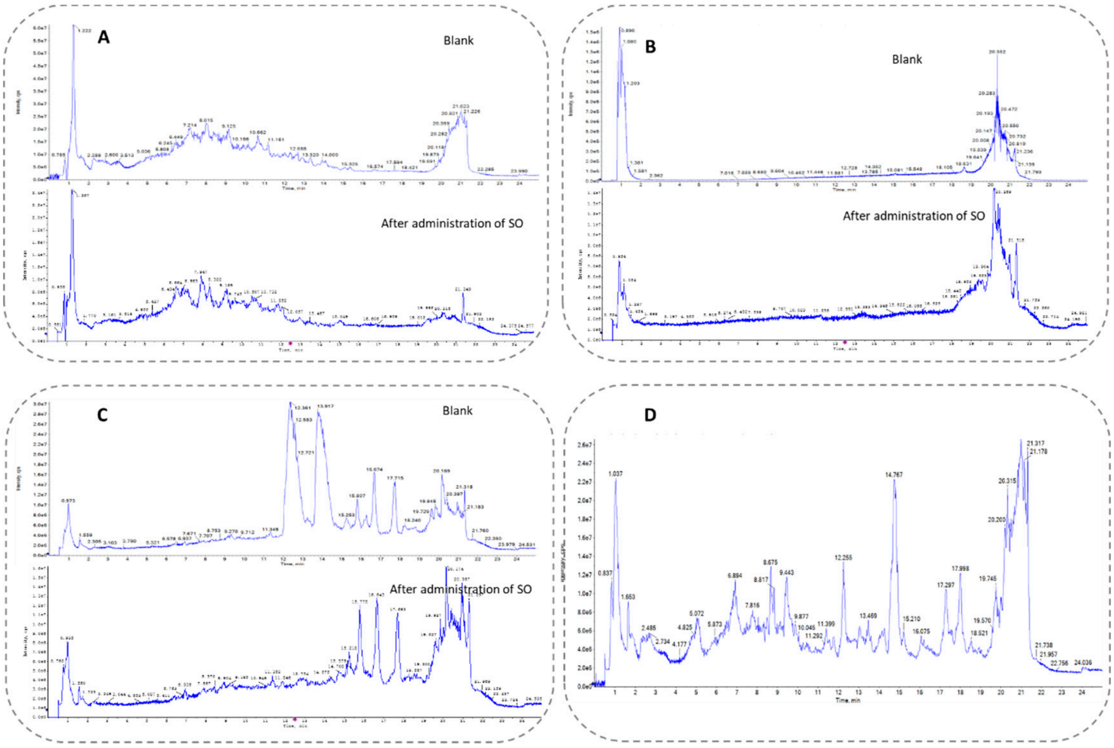

Figure S1. Total ion chromatograms of: (**A**) urine; (**B**) plasma; (**C**) feces, before and after administration of SO. (**D**) SO extract alone.

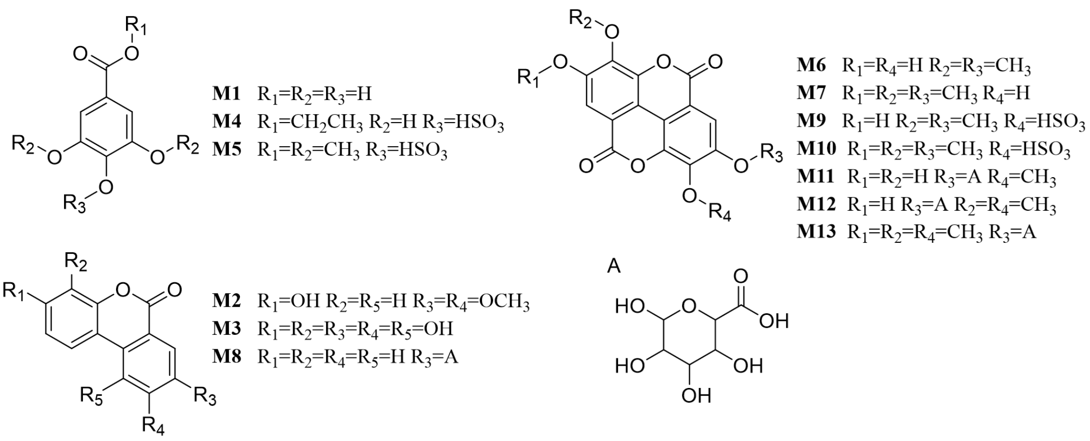

Figure S2. Metabolites of ellagic acids (EAs) in vivo.

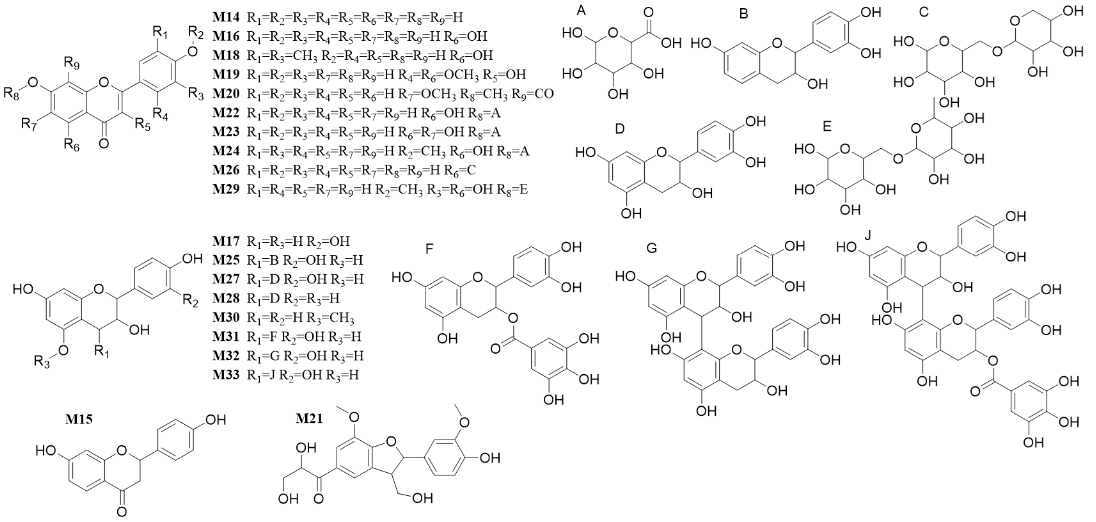

Figure S3. Metabolites of flavonoids in vivo.

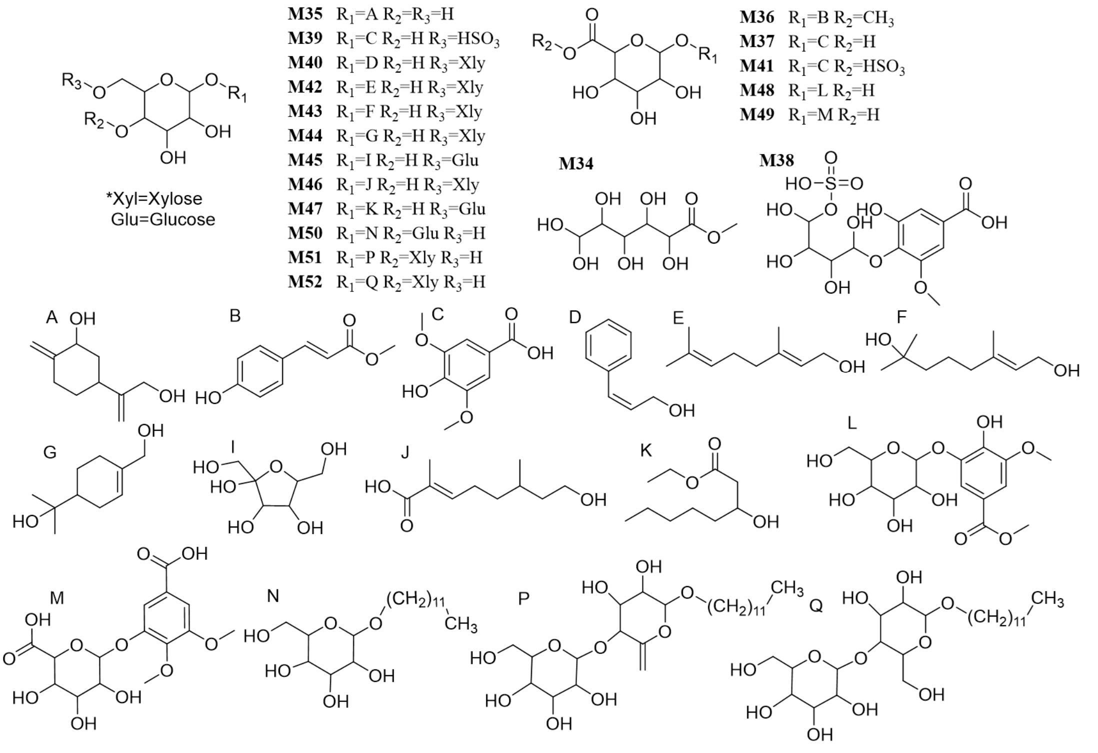

Figure S4. Metabolites of glycosides in vivo.

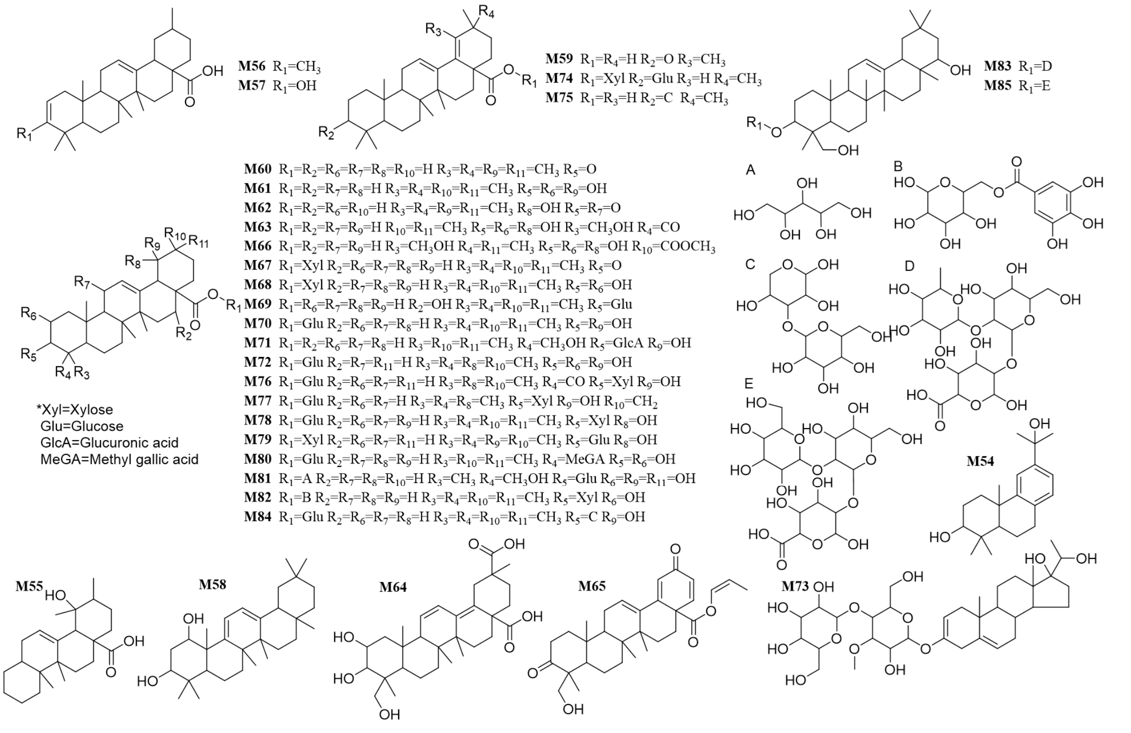

Figure S5. Metabolites of saponins in vivo.

Table S1. Structures of the tentatively characterized compounds in *Sanguisorba officinalis* L.

Metabolites	Class	Ion type	Molecular Formula	Molecular ion	Retention Time (min)	ppm	Fragments	References
**M1**	EA	$[M–H]^-$	$C_7H_6O_5$	169.0143	2.555	3.5	125.0245, 107.0136, 97.0295, 79.0188	[1]
**M2**	EA	$[M–H]^-$	$C_{15}H_{12}O_5$	271.0618	9.940	4.2	243.0564, 227.0609, 109.0653	[2]
**M3**	EA	$[M–H]^-$	$C_{13}H_8O_7$	275.0201	1.532	3.3	195.0663, 165.0559, 151.0769	[3]
**M4***	EA	$[M–H]^-$	$C_9H_{10}O_8S$	277.0022	7.146	1.4	197.0457, 182.0226, 166.9989	–
**M5**	EA	$[M–H]^-$	$C_{10}H_{12}O_8S$	291.0185	9.400	3.5	211.0606, 181.0137, 153.0191	[4]
**M6**	EA	$[M–H]^-$	$C_{16}H_{10}O_8$	329.0307, 331.0450	11.422	2.9	315.0104, 298.9837, 285.0048	[5]
**M7**	EA	$[M+H]^+$	$C_{17}H_{12}O_8$	345.0602	16.093	–2.4	330.0369, 313.0346, 285.0395	[6]

**M8**	EA	$[M-H]^-$	$C_{19}H_{16}O_9$	387.0711	11.851	–1.3	211.0614, 181.0145, 175.0252, 153.0194	[2]
**M9***	EA	$[M-H]^-$	$C_{16}H_{10}O_{11}S$	408.9878	16.918	3.0	329.0305, 314.0069, 298.9836, 270.9888	–
**M10***	EA	$[M-H]^-$	$C_{17}H_{12}O_{11}S$	423.0024	12.678	0.5	343.0461, 328.0225, 312.9991, 297.9760	–
**M11***	EA	$[M-H]^-$	$C_{21}H_{16}O_{14}$	491.0471	9.384	1.8	315.0143, 299.9911, 96.9597	–
**M12**	EA	$[M-H]^-$	$C_{22}H_{18}O_{14}$	505.0617	9.805	–0.3	329.0294, 314.0056	[7]
**M13**	EA	$[M-H]^-$	$C_{23}H_{20}O_{14}$	519.0763	11.761	–2.3	343.0454, 328.0218, 312.9984, 297.9747	[8]
**M14**	Flavonoids	$[M+H]^+$	$C_{15}H_{10}O_4$	255.0651	12.614	–2.5	237.0548, 227.0702, 199.0752, 153.0692, 137.0236	[9]
**M15**	Flavonoids	$[M+H]^+$	$C_{15}H_{12}O_4$	257.0802	12.748	–4.6	239.0686, 163.0376, 137.0223, 95.0481	[10]

**M16**	Flavonoids	$[M+H]^+$	$C_{15}H_{10}O_5$	271.0593	14.919	–4.9	253.0492, 215.0692, 197.0589, 169.0639, 153.0173	[11]
**M17**	Flavonoids	$[M+H]^+$	$C_{15}H_{14}O_6$	291.0869	6.915	0.1	147.0439, 139.0387, 123.0438, 119.0493	[12]
**M18***	Flavonoids	$[M–H]^–$	$C_{17}H_{14}O_7$	329.0664	15.031	0.8	299.0192, 271.0244, 259.1539, 227.0344, 161.0240	–
**M19**	Flavonoids	$[M+H]^+$	$C_{17}H_{14}O_7$	331.0814	15.209	–1.1	253.0847, 211.0745	[13]
**M20**	Flavonoids	$[M+CH_3OH+H]^+$	$C_{19}H_{16}O_6$	373.1279	11.856	–2.2	313.1087, 271.0968	[14]
**M21**	Flavonoids	$[M+NH_4]^+$	$C_{20}H_{22}O_8$	408.1645	12.136	–3.2	373.1283, 313.1057, 281.0795	[15]
**M22**	Flavonoids	$[M+H]^+$	$C_{21}H_{18}O_{10}$	431.0968	8.772	–2.4	255.0648, 199.0752, 181.0650	[16]
**M23**	Flavonoids	$[M–H]^–$	$C_{21}H_{18}O_{11}$	445.0768	10.284	–0.6	269.0455, 175.0249	[17]

**M24**	Flavonoids	$[M+H]^+$	$C_{22}H_{20}O_{11}$	461.1069	8.993	–3.2	285.0756	[18]
**M25***	Flavonoids	$[M+H]^+$	$C_{30}H_{26}O_{11}$	563.1549	8.852	–0.8	435.1078, 423.1078, 411.1065, 393.0963, 283.0597, 165.0547	–
**M26**	Flavonoids	$[M+H]^+$	$C_{26}H_{28}O_{14}$	565.1540	8.706	–3.0	547.1404, 529.1314, 511.1216, 184.1116, 379.0805, 325.0692	[19]
**M27**	Flavonoids	$[M+H]^+$	$C_{30}H_{26}O_{12}$	579.1491	6.792	–1.9	427.1038, 409.0925, 301.0712, 287.0555, 275.0556	[20]
**M28**	Flavonoids	$[M+Na]^+$	$C_{30}H_{26}O_{11}$	585.1375	8.423	0.4	433.0889, 415.0787, 303.0729, 277.0304, 153.0186	[21]
**M29**	Flavonoids	$[M+Na]^+$	$C_{30}H_{26}O_{12}$	601.1325	6.770	0.5	449.0847, 431.0736, 311.0523, 279.0266	[22]
**M30**	Flavonoids	$[2M+K]^+$	$C_{16}H_{16}O_5$	615.1634	8.309	0.2	–	[23]
**M31**	Flavonoids	$[M+H]^+$	$C_{37}H_{30}O_{16}$	731.1602	8.423	–1.4	579.1129, 411.1066, 331.0443, 303.0498, 291.0859, 287.0547, 163.0389, 153.0179	[20]

**M32**	Flavonoids	$[M+H]^+$	$C_{45}H_{38}O_{18}$	867.2136	7.049	–0.05	715.1645, 577.1329, 427.1014, 407.0754, 289.0702, 247.0597, 163.0388	[24]
**M33**	Flavonoids	$[M+H]^+$	$C_{52}H_{42}O_{22}$	1019.2249	7.901	0.3	867.1784, 729.1450, 697.1542, 579.1144, 559.1233, 411.1073	[25]
**M34**	Glycosides	$[M–H]^–$	$C_7H_{14}O_8$	225.0618	1.058	3.4	113.0239, 101.0245, 89.0243, 71.0139	[26]
**M35**	Glycosides	$[M+NH_4]^+$	$C_{16}H_{26}O_7$	348.2020	13.257	–0.7	147.0811, 137.1327, 123.1158, 95.0850	[27]
**M36**	Glycosides	$[M–H]^–$	$C_{17}H_{20}O_9$	367.1047	0.953	4.8	307.0840, 277.0735	[28]
**M37**	Glycosides	$[M–H]^–$	$C_{15}H_{18}O_{11}$	373.0769	1.312	–0.5	197.0451, 182.0215	[29]
**M38***	Glycosides	$[M–H]^–$	$C_{12}H_{16}O_{13}S$	399.0244	3.017	2.7	301.0579, 241.0363, 211.0258, 169.0145	–
**M39**	Glycosides	$[M–H]^–$	$C_{15}H_{20}O_{13}S$	439.0564	6.784	4.0	359.0992, 241.0028, 197.0453, 182.0219	[30]

**M40**	Glycosides	$[M+NH_4]^+$	$C_{20}H_{28}O_{10}$	446.2024	9.243	–0.5	241.0705,117.0698, 115.0541, 91.0543	[31]
**M41**	Glycosides	$[M–H]^–$	$C_{15}H_{18}O_{14}S$	453.0340	1.201	0.2	373.0778, 277.0022, 197.0453	–
**M42**	Glycosides	$[M+Na]^+$	$C_{21}H_{36}O_{10}$	466.2642 471.2202	12.269	–0.8	259.0816, 241.0709, 223.0602, 163.0601	[32]
**M43**	Glycosides	$[M+NH_4]^+$	$C_{21}H_{38}O_{11}$	484.2752 489.2301	8.682	–1.2	295.1027, 259.0818, 241.0711, 163.0601	[33]
**M44**	Glycosides	$[M+Na]^+$	$C_{21}H_{36}O_{11}$	487.2150	9.460	–1.1	355.1725, 335.0947	[34]
**M45**	Glycosides	$[M–H]^–$	$C_{18}H_{32}O_{16}$	503.1610	1.047	–1.5	353.1069, 263.0763, 131.0351	[22]
**M46***	Glycosides	$[M+Na]^+$	$C_{21}H_{36}O_{12}$	503.2100	8.509	–0.9	335.0957, 317.0845, 229.0684, 209.1153, 185.0425	–
**M47**	Glycosides	$[M–H]^–$	$C_{22}H_{40}O_{13}$	511.2409	8.547	3.6	333.1929, 311.0991, 293.0882, 211.0672, 179.0565	[35]

**M48***	Glycosides	$[M+Na]^+$	$C_{21}H_{28}O_{16}$	559.1268	6.767	–1.3	383.0945, 221.0418, 185.0418	–
**M49***	Glycosides	$[M+K]^+$	$C_{21}H_{26}O_{17}$	589.0802	1.275	–0.8	413.0477, 237.0162, 214.9946	–
**M50**	Glycosides	$[M+H]^+$	$C_{30}H_{56}O_{16}$	673.3621	17.982	–3.8	511.3095, 493.2995	[36]
**M51***	Glycosides	$[M+H]^+$	$C_{35}H_{62}O_{19}$	787.3938	17.333	–2.5	671.3464, 625.3409	–
**M52***	Glycosides	$[2M+Na]^+$	$C_{15}H_{18}O_{11}$	771.1559	7.957	–4.8	397.0731, 221.0415, 199.0212	–
**M53***	Glycosides	$[M+H]^+$	$C_{35}H_{64}O_{20}$	805.4052	14.549	–2.1	689.3567, 643.3521, 493.3004	–
**M54**	Saponins	$[M+H]^+$	$C_{20}H_{30}O_2$	303.2319	21.010	–1.7	145.1018, 131.0862, 105.0704	[37]
**M55***	Saponins	$[M+H]^+$	$C_{23}H_{36}O_3$	361.2731	17.917	–3.2	343.2634, 201.1636, 137.0961	–

**M56***	Saponins	$[M+H]^+$	$C_{30}H_{44}O_2$	437.3410	14.784	–2.2	391.3353, 201.1635	–
**M57***	Saponins	$[M+H]^+$	$C_{29}H_{44}O_3$	441.3364	16.395	–1.1	423.3244, 351.2704	[38]
**M58**	Saponins	$[M+H]^+$	$C_{30}H_{48}O_2$	441.3711	21.028	–4.8	423.3610, 405.3508, 369.3138, 247.2049, 203.1785	[39]
**M59**	Saponins	$[M+H]^+$	$C_{30}H_{44}O_3$	453.3373	14.119	0.9	435.3261, 407.3320, 335.2726, 201.1645	[40]
**M60**	Saponins	$[M+H]^+$	$C_{30}H_{46}O_3$	455.3528	14.763	–0.6	437.3411, 409.3463, 391.3357, 201.1636	[41]
**M61***	Saponins	$[M–H]^–$	$C_{30}H_{48}O_5$	487.3423	19.449	–0.1	373.1829	–
**M62**	Saponins	$[M+NH_4]^+$	$C_{30}H_{44}O_5$	502.3530	20.878	–0.5	467.3152, 449.3069, 421.3106, 403.2994	[42]
**M63**	Saponins	$[M–H]^–$	$C_{30}H_{46}O_7$	517.3181	13.307	3.0	473.3279, 455.3195, 407.2962, 365.0313	[43]
**M64**	Saponins	$[M+NH_4]^+$	$C_{30}H_{44}O_7$	534.3428	18.326	–0.5	487.3044, 471.3112, 435.2853, 409.3093, 365.2845	[44]

**M65***	Saponins	$[M+FA–H]^-$	$C_{31}H_{40}O_5$	537.2879	18.305	4.9	501.3200, 491.2321	–
**M66***	Saponins	$[M–H]^-$	$C_{31}H_{48}O_8$	547.3293	16.661	4.0	501.3237, 483.3118, 439.3231	–
**M67**	Saponins	$[M+H]^+$	$C_{35}H_{54}O_7$	587.3943	14.741	–0.8	455.3508, 437.3415, 409.3427	[45]
**M68**	Saponins	$[M+H]^+$	$C_{35}H_{56}O_8$	605.4055	14.784	0.3	455.3513, 437.3418	[46]
**M69**	Saponins	$[M+NH_4]^+$	$C_{36}H_{58}O_9$	652.4413	17.982	–1.7	473.3624, 455.3507, 437.3411, 391.3358, 163.1481	[47]
**M70**	Saponins	$[M+Na]^+$	$C_{36}H_{58}O_9$	657.3965	17.982	–2.1	495.3445, 185.0420	[48]
**M71**	Saponins	$[M–H]^-$	$C_{36}H_{56}O_{11}$	663.3758	13.392	2.0	617.3656, 487.3443, 175.0243	[49]
**M72**	Saponins	$[M+NH_4]^+$	$C_{36}H_{58}O_{10}$	668.4364	14.271	–1.4	489.4364, 471.3467, 407.3304	[50]
**M73**	Saponins	$[M+CH_3OH+H]^+$	$C_{34}H_{54}O_{13}$	703.3901	17.982	–0.5	657.3850, 477.3221, 185.0303	[51]

**M74***	Saponins	$[M+NH_4]^+$	$C_{41}H_{64}O_{12}$	766.4732	17.312	–1.2	455.3526, 437.3405, 409.3471	–
**M75**	Saponins	$[M+Na]^+$	$C_{41}H_{64}O_{12}$	771.4285	17.312	–1.4	609.3758, 565.3870, 185.0424	[52]
**M76**	Saponins	$[M+H]^+$	$C_{41}H_{64}O_{14}$	781.4371	12.549	0.4	649.3947, 487.3421, 469.3316, 423.3255	[53]
**M77**	Saponins	$[M+NH4]^+$	$C_{41}H_{64}O_{13}$	782.4690	15.236	– 0.08	603.3898, 557.3835, 471.3477, 453.3365, 425.3426, 407.3312, 180.0869	[54]
**M78**	Saponins	$[M+NH_4]^+$	$C_{41}H_{66}O_{13}$	784.4832	14.699	–1.9	455.3509, 437.3410, 409.3459	[55]
**M79**	Saponins	$[M+Na]^+$	$C_{41}H_{66}O_{13}$	789.4384	14.741	–2.1	627.3859, 185.0422	[56]
**M80**	Saponins	$[M+Na]^+$	$C_{43}H_{62}O_{14}$	825.4022	14.592	–1.8	641.3898	[57]
**M81***	Saponins	$[M+H]^+$	$C_{40}H_{66}O_{18}$	835.4316	14.522	–1.3	789.4263, 627.3727, 537.3434, 477.3229	–

**M82**	Saponins	$[M+NH_4]^+$	$C_{48}H_{70}O_{17}$	936.4963	15.039	0.7	455.3523, 437.3416, 391.3366, 153.0182	[58]
**M83**	Saponins	$[M+H]^+$	$C_{48}H_{78}O_{18}$	943.5229	17.539	–3.9	797.4654, 599.3913, 441.3704, 423.3600	[59]
**M84**	Saponins	$[M+NH_4]^+$	$C_{47}H_{76}O_{18}$	946.5385	14.763	1.0	605.4053, 455.3520, 437.3423, 409.3477, 163.0606	[60]
**M85**	Saponins	$[M+H]^+$	$C_{48}H_{78}O_{19}$	959.5192	17.208	–2.5	797.4646, 617.4027, 599.3929, 441.3709, 423.3609, 163.0591	[61]
**M86**	Others	$[M–H]^–$	$C_7H_6O_2$	121.0292	7.980	2.0	93.0335	[62]
**M87***	Others	$[M–H]^–$	$C_5H_6O_4$	129.0193	1.123	4.0	111.0088, 87.0089, 85.0296, 67.0191	–
**M88***	Others	$[M+H]^+$	$C_{10}H_{16}O$	153.1278	10.001	–0.9	105.0697, 91.0542, 79.0537	–
**M89**	Others	$[M+NH_4]^+$	$C_6H_8N_2O_2$	158.0923	0.924	–4.1	70.0651	[63]

**M90**	Others	$[M+H]^+$	$C_{11}H_{14}O$	163.1119	8.750	–2.4	–	[64]
**M91**	Others	$[M+H]^+$	$C_{10}H_{10}O_3$	179.0704	7.208	–2.3	164.0470, 91.0538, 77.0387	[65]
**M92***	Others	$[M+H]^+$	$C_{11}H_{16}O_2$	181.1220	8.750	–4.7	152.0598, 121.1001, 93.0694, 79.0542	–
**M93**	Others	$[M–H]^–$	$C_{10}H_{10}O_4$	193.0504	9.959	1.6	133.0296	[66]
**M94**	Others	$[M–H]^–$	$C_{12}H_{18}O_4$	225.1127	8.621	0.07	97.0658, 59.0137	[67]
**M95**	Others	$[M+H]^+$	$C_{14}H_{12}O_3$	229.0856	20.394	–3.7	151.0387, 105.0337	[68]
**M96**	Others	$[M+H]^+$	$C_{14}H_{24}O_3$	241.1802	20.851	–0.7	93.0706, 83.0497	[69]
**M97**	Others	$[M+H]^+$	$C_{15}H_{18}O_6$	295.1172	19.856	–3.3	133.0645, 107.0840, 105.0685	[70]

**M98***	Others	$[M+H]^+$	$C_{14}H_{30}O_4S$	295.1930	20.167	–2.6	193.1251, 189.1633, 133.0642, 67.0542	–
**M99**	Others	$[M+H]^+$	$C_{19}H_{30}O_3$	307.2263	20.649	–3.3	165.1270, 109.0648, 81.0697	[71]
**M100***	Others	$[M+H]^+$	$C_{18}H_{34}O_4$	315.2533	20.872	–0.7	185.1178, 139.1121, 121.1017	–
**M101***	Others	$[M–H]^–$	$C_{22}H_{36}O_2$	331.2631	20.871	–1.8	287.2723	–
**M102**	Others	$[M+H]^+$	$C_{20}H_{22}O_8$	391.1389	11.769	–1.0	195.0656, 151.0397, 137.0601	[16]
**M103***	Others	$[M+Na]^+$	$C_{28}H_{41}NO_4$	478.2914	20.177	–4.0	337.2717, 306.2776	–
**M104**	Others	$[M+Na]^+$	$C_{30}H_{45}NO_4$	506.3228	20.918	–3.6	365.3030, 240.0987	[72]

*: structures were reported for the first time.

References

1 Awantika S., Vikas B., Sunil K., Kulwant R.S., Brijesh K. Profiling of Gallic and Ellagic Acid Derivatives in Different Plant Parts of Terminalia arjuna by HPLC–ESI–QTOF–MS/MS. Nat. Prod. Commun. 2016; 11(2): 239–244.

2 Shahraki A., Ebrahimi A. Ellagitannin derivatives and some conjugated metabolites: aqueous–DMSO proton affinities and acidity constants. J. Struct. Chem. 2019; 30(4): 1343–1351. https://doi.org/10.1007/s11224–019–1284–8.

3 Watanabe H., Kishino S., Kudoh M., Yamamoto H., Ogawa J. Evaluation of electron–transferring cofactor mediating enzyme systems involved in urolithin dehydroxylation in Gordonibacter urolithinfaciens DSM 27213. J. Biosci. Bioeng. 2020; 129(5): 552–557. https://doi.org/10.1016/j.jbiosc.2019.11.014.

4 Kato Y., Kawai M., Kawai S., Okano Y., Rokkaku N., A. Ishisaka, K. Murota, T. Nakamura, Y. Nakamura, S. Ikushiro. Dynamics of the Cellular Metabolism of Leptosperin Found in Manuka Honey. J. Agric. Food Chem. 2019; 67(39): 10853–10862. https://doi.org/10.1021/acs.jafc.9b03894.

5 Li D.X., Chou G.X., Wang Z.T. Chemical constituents from n–butanol fraction of *Senecio solidagineus*. Chin. J. Pharm. 2014; 45(7): 626–628.

6 Balde M.A., Tuenter E., Matheeussen A., Traore M.S., Cos P., L. Maes, A. Camara, M.S.T. Diallo, E.S. Balde, A.M. Balde, L. Pieters, K. Foubert. Bioassay–guided isolation of antiplasmodial and antimicrobial constituents from the roots of *Terminalia albida*. J. Ethnopharmacol. 2021; 267: 113624. https://doi.org/10.1016/j.jep.2020.113624.

7 Ma J.Y., Zhou X., Fu J., He C.Y., Feng R., Huang M., J.W. Shou, Z.X. Zhao, X.Y. Li, L. Zhang, Y.C. Chen, Y. Wang. In Vivo Metabolite Profiling of a Purified Ellagitannin Isolated from *Polygonum capitatum* in Rats. Molecules. 2016; 21(9). https://doi.org/10.3390/molecules21091110.

8 Ayoub N.A. A trimethoxyellagic acid glucuronide from *Conocarpus erectus* leaves: isolation, characterization and assay of antioxidant capacity. Pharm. Biol. 2010; 48(3): 328–332. https://doi.org/10.3109/13880200903131567.

9 Silva H.C., Pinto F.C.L., Sousa A.F., Pessoa O.D.L., M.T.S. Trevisan, G.M.P. Santiago. Chemical constituents and acetylcholinesterase inhibitory activity from the stems of Bauhinia pentandra. Nat. Prod. Res. 2020: 1–5. https://doi.org/10.1080/14786419.2020.1752206.

10 Wang D.W., Xiao C.J., Qiu L, Tian X.Y., Dong X., Jiang B. Two new 8–isopentenyl isoflavane derivatives from *Astragalus dolichochaete* diels. Nat. Prod. Res. 2021; 35(8): 1323–1330. https://doi.org/10.1080/14786419.2019.1647426.

11 Ifeanacho M.O., Ogunwa S.C., Amadi P.U. Phytochemical Composition of *Vitex doniana*. Anal. Chem. Lett. 2020; 9(6): 863–875. https://doi.org/10.1080/22297928.2020.1722221.

12 Achili I., Amrani A., Bensouici C., Gul F., M. Altun, I. Demirtas, D. Zama, F. Benayache, S. Benayache. Chemical Constituents, Antioxidant, Anticholinesterase and Antiproliferative Effects of Algerian *Pistacia atlantica* Desf. Extracts. Recent. Pat. Food Nutr. Agric. 2020; 11(3): 249–256. https://doi.org/10.2174/2212798411666200207101502.

13 Zhang P., Feng Z., Wang Y. Flavonoids, including an unusual flavonoid–Mg2+ salt, from roots of *Cudrania cochinchinensis*. Phytochemistry. 2005; 66(23): 2759–65. https://doi.org/10.1016/j.phytochem.2005.09.015.

14 Chen J.X., Leng H.Q., Duan Y.X., Zhao W., Yang G.Y., Guo Y.D., Chen Y.K., Hu Q.F. Three new flavonoids from the leaves of oriental tobacco and their cytotoxicity. Phytochem. Lett. 2013; 6(1): 144–147. https://doi.org/10.1016/j.phytol.2012.12.001.

15 Liu Q.B., Huang X.X., Bai M., Chang X.B., Yan X.J., Zhu T., Zhao W., Peng Y., Song S.J. Antioxidant and anti–inflammatory active dihydrobenzofuran neolignans from the seeds of *Prunus tomentosa*. J. Agric. Food Chem. 2014; 62(31): 7796–7803. https://doi.org/10.1021/jf502171z.

16 Flamini G., Pardini M., Morelli I., Ertugrul K., Dural H., Bagci Y., Kargioglu M. Flavonoid glycosides from *Centaurea pseudoscabiosa* subsp. *pseudoscabiosa* from Turkey. Phytochemistry 2002; 61: 433–437. https://doi.org/10.1016/S0031–9422(02)00229–7.

17 Baygildieva D.I., Baygildiev T.M., Stavrianidi A.N., Shpigun O.A., Rodin I.A. Simultaneous Determination of Wogonin, Scutellarin, Baicalin, and Baicalein in Extracts from *Scutellariae Baicalensis* by High–Performance Liquid Chromatography with Tandem Mass Spectrometry. J. Anal. Chem. 2019; 73(14): 1317–1322. https://doi.org/10.1134/s1061934818140022.

18 Salama M.M., Ezzat S.M., Sleem A.A. A New Hepatoprotective Flavone Glycoside from the Flowers of *Onopordum alexandrinum* Growing in Egypt. Zeitschrift fuer Naturforschung, C: Journal of Biosciences. 2011; 66: 251–259. https://doi.org/10.1515/znc–2011–5–608.

19 Feng C.Y., Wu Q ., Yin D.D., Li B., Li S.S., Tang Z.Q., Xu Y.J., Wang L.S. Determination of xanthones and flavonoids of methanol extracts obtained from different parts of the plants of three Gentianaceae species. J. Pharm. Biomed. Anal. 2018; 161: 455–463. https://doi.org/10.1016/j.jpba.2018.08.059.

20 Zhang H.W., Yu M., Jia H.M., Zhang T., H. Shang, M.L. Zhang, Z. Zhu, Z.M. Zou. Comprehensive identification of potential antioxidant components in the aerial parts of *Polygonum chinense* L. var. *hispidum* using ultra high performance liquid chromatography coupled with quadrupole time–of–flight mass spectrometry. J. Sep. Sci. 2020; 43(12): 2380–2392. https://doi.org/10.1002/jssc.201901291.

21 Bicker J., Petereit F., Hensel A. Proanthocyanidins and a phloroglucinol derivative from *Rumex acetosa* L. Fitoterapia. 2009; 80(8): 483–495. https://doi.org/10.1016/j.fitote.2009.08.015.

22 Fu Y., Shan M., Hu M., Jiang Y., Chen P., Chi Y., S. Yu, L. Zhang, Q. Wu, F. Zhang, Z. Mao. Chemical profiling of Banxia–Baizhu–Tianma decoction by ultra–fast liquid chromatography with tandem mass spectrometry. J. Pharm. Biomed. Anal. 2019; 174: 595–607. https://doi.org/10.1016/j.jpba.2019.06.030.

23 Liao S.G., Zhang B.L., Wu Y., Yue J.M. New Phenolic Components from *Daphne giraldii*. Helvetica Chimica Acta. 2005; 88: 2873–2878. https://doi.org/10.1002/hlca.200590230.

24 Khallouki F., Haubner R., Hull W. E., Erben G., Spiegelhalder B., Bartsch H., Owen R.W. Isolation, purification and identification of ellagic acid derivatives, catechins, and procyanidins from the root bark of *Anisophyllea dichostyla* R. Br. Food Chem. Toxicol. 2007; 45(3): 472–485. https://doi.org/10.1016/j.fct.2006.09.011.

25 Fitzpatrick D.F., Bing B., Maggi D.A., Fleming R.C., O'malley R.M. Vasodilating Procyanidins Derived from Grape Seeds. Ann. N. Y. Acad. Sci. 2002; 957: 78–89. https://doi.org/10.1111/j.1749–6632.2002.tb02907.x.

26 Kim S.P., Moon E., Nam S.H., Friedman M. Composition of Herba Pogostemonis water extract and protection of infected mice against *Salmonella* Typhimurium–induced liver damage and mortality by stimulation of innate immune cells. J. Agric. Food Chem. 2012; 60(49): 12122–12130. https://doi.org/10.1021/jf304466t.

27 Toshmatov Z.O., Li J., Eshbakova K.A., Tang D., X.L. Xin, H. Akber Aisa. New monoterpene glucosides from *Dracocephalum komarovi* and their anti–inflammatory activity. Phytochem. Lett. 2019; 33: 102–105. https://doi.org/10.1016/j.phytol.2019.08.005.

28 Fang X., Fu Z., Zhang H., Xu H.X., Liang S. Chemical Constituents of *Garcinia yunnanensis* and Their Scavenging Activity Against DPPH Radicals. Chem. Nat. Compd. 2018; 54(2): 232–234. https://doi.org/10.1007/s10600–018–2310–6.

29 Ancillotti C., Ulaszewska M., Mattivi F., Del Bubba M. Untargeted Metabolomics Analytical Strategy Based on Liquid Chromatography/Electrospray Ionization Linear Ion Trap Quadrupole/Orbitrap Mass Spectrometry for Discovering New Polyphenol Metabolites in Human Biofluids after Acute Ingestion of *Vaccinium myrtillus* Berry Supplement. J. Am. Soc. Mass Spectrom. 2019; 30(3): 381–402. https://doi.org/10.1007/s13361–018–2111–y.

30 He L.L., Zhang Z.F., Liu Y., Chen D.Q., Yuan M.H., G.T. Dong, P. Luo, Z.G. Yan. Rapid discrimination of raw and sulfur–fumigated *Smilax glabra* based on chemical profiles by UHPLC–QTOF–MS/MS coupled with multivariate statistical analysis. Food Res. Int. 2018; 108: 226–236. https://doi.org/10.1016/j.foodres.2018.03.047.

31 Ma C.Y., Tang J., Wang H.X., Gu X.H., Tao G.J. Simultaneous Determination of Six Active Compounds in *Rhodiola* L. by RP–LC. Chromatographia. 2008; 67(5): 383–388. https://doi.org/10.1365/s10337–008–0539–0.

32 Sun W., Zhang Z.L., Liu X., Zhang S., He L., Wang Z., Wang G.S. Terpene glycosides from the roots of *Sanguisorba officinalis* L. and their hemostatic activities. Molecules. 2012; 17(7): 7629–7636. https://doi.org/10.3390/molecules17077629.

33 Ji C.J., Tan N.H., Fu J., Zhang Y.M., He M. Monoterpene disaccharide glycosides from *Rodgersia pinnata*. Yunnan Zhiwu Yanjiu. 2004; 26(4): 465–470.

34 Daikonya A., Kitanaka S. Constituents isolated from the roots of Rhodiola sacra S. H. Fu. Nippon Shokuhin Kagaku Gakkaishi. 2011; 18(3): 183–190.

35 Mayorga H., Duque C., Knapp H., Winterhalter P. Hydroxyester disaccharides from fruits of cape gooseberry (*Physalis peruviana*). Phytochemistry. 2002; 59: 439–445. https://doi.org/10.1016/S0031–9422(01)00467–8.

36 Marcon F., Moreau V., Helle F., Thiebault N., Djedaini–Pilard F., Mullie C. *β*–Alkylated oligomaltosides as new alternative preservatives: antimicrobial activity, cytotoxicity and preliminary investigation of their mechanism of action. J. Appl. Microbiol. 2013; 115(4): 977–86. https://doi.org/10.1111/jam.12301.

37 Guo K., Liu Y.C., Liu Y., Luo S.H., W.Y. Li, X.N. Li, S.H. Li. Diversified abietane family diterpenoids from the leaves of *Leucosceptrum canum* and their cytotoxic activity. Phytochemistry. 2019; 157: 43–52. https://doi.org/10.1016/j.phytochem.2018.10.010.

38 Pu X.Y., Zhou J. A new triterpenoid of *psammosilene tunicoides*. Acta Botanica Yunnanica. 1987; 9(3): 369–370.

39 Zhang Y.W., Lin H., Bao Y.L., Wu Y., Yu C.L., Huang Y.X., Li Y.X. A new triterpenoid and other constituents from the stem bark of *Juglans mandshurica*. Biochem. Syst. Ecol. 2012; 44: 136–140. https://doi.org/10.1016/j.bse.2012.04.015.

40 Ali M., Bhutanni K.K., Srivastava T.N. Triterpenoids from *Symplocos racemosa* bark. Phytochemistry. 1990; 29(11): 3601–3604. https://doi.org/10.1016/0031–9422(90)85284–M.

41 Li R., Nie L., Wang Q., Ouyang D., Zhang L., Yuan Y., Hong Y., Wang J., Hu X. Phytochemical constituents, chemotaxonomic significance and anti–arthritic effect of *Eucommia ulmoides* Oliver staminate flowers. Nat. Prod. Res. 2020: 1–5. https://doi.org/10.1080/14786419.2020.1858411.

42 Pu Z.H., Tang H.Q., Long N.N., Qiu M., M.X. Gao, F.H. Sun, M. Dai. Assessment of the anti–virulence potential of extracts from four plants used in traditional Chinese medicine against multidrug–resistant pathogens. Complement. Med. Ther. 2020; 20(1): 318. https://doi.org/10.1186/s12906–020–03114–z.

43 Pérez A.J., Pecio U., Kowalczyk M., Kontek R., Gajek G., Stopinsek L., Mirt I., Oleszek W., Stochmal A. Triterpenoid components from oak heartwood (*Quercus robur*) and their potential health benefits. J. Agric. Food Chem. 2017; 65(23): 4611–4623. https://doi.org/10.1021/acs.jafc.7b01396.

44 Ma X.P., Lou H.Y., Zhang W.F., Song J.R., Y. Li, W.D. Pan. Triterpenoids from *Phytolacca acinosa*. Chem. Nat. Compd. 2019; 55(2): 292–295. https://doi.org/10.1007/s10600–019–02670–2.

45 Li J.F., Chen S.J., Zhao Y., Li J.X. Glycoside modification of oleanolic acid derivatives as a novel class of anti–osteoclast formation agents. Carbohydr. Res. 2009; 344(5): 599–605. https://doi.org/10.1016/j.carres.2009.01.019.

46 Yadava R.N. A novel triterpenoid saponin from the leaves of *Majorana hortensis* (Moench). Advances in Experimental Medicine and Biology. 1996; 405: 223–230.

47 Guenne S., Ouattara N., Ouedraogo N., Ciobica A., A. Hilou, M. Kiendrebeogo. Phytochemistry and neuroprotective effects of *Eclipta alba* (L.) Hassk. J. Complement. Integr. Med. 2019; 17(1): 1–5. https://doi.org/10.1515/jcim–2019–0026.

48 Yang B., Li H., Ruan Q.F., Xue Y.Y., Cao D., Zhou X.H., Jiang S.Q., Yi T., Jin J., Zhao Z.X. A facile and selective approach to the qualitative and quantitative analysis of triterpenoids and phenylpropanoids by UPLC/Q–TOF–MS/MS for the quality control of *Ilex rotunda*. J. Pharm. Biomed. Anal. 2018; 157: 44–58. https://doi.org/10.1016/j.jpba.2018.05.002.

49 Tan Q., Qiu M., Cao D., Xiong T., Zhang L., Zhou L., Rong L., J. Zhou, J. Jin, Z. Zhao. Triterpenoids with Antiplatelet Aggregation Activity from the Roots of *Ilex pubescens*. Planta Med. 2017; 83(9): 797–804. https://doi.org/10.1055/s–0042–123708.

50 Wu Y.S., Shi L., Liu X.G., Li W., Wang R., S. Huang, Y. Li, D.L. Yan, H.Y. Wang, Y. Tian, Y.M. Chen, H. Yang. Chemical profiling of *Callicarpa nudiflora* and its effective compounds identification by compound–target network analysis. J. Pharm. Biomed. Anal. 2020; 182: 113110. https://doi.org/10.1016/j.jpba.2020.113110.

51 Wang L., Yin Z.Q., Zhang Q.W., Zhang X.Q., Zhang D.M., Liu K., Li Y.L., Yao X.S., Ye W.C. Five new C21 steroidal glycosides from *Periploca sepium*. Steroids. 2011; 76(3): 238–243. https://doi.org/10.1016/j.steroids.2010.11.004.

52 Wu X., Wang L., Wang G.C., Wang H., Dai Y., Yang X.X., Ye W.C., Li Y.L. Triterpenoid saponins from rhizomes of *Paris polyphylla* var. *yunnanensis*. Carbohydr. Res. 2013; 368: 1–7. https://doi.org/10.1016/j.carres.2012.11.027.

53 Wu P., Gao H., Liu J.X., Liu L., Zhou H., Liu Z.Q. Triterpenoid saponins with anti–inflammatory activities from *Ilex pubescens* roots. Phytochemistry. 2017; 134: 122–132. https://doi.org/10.1016/j.phytochem.2016.11.012.

54 Wu Z.J., Ouyang M.A., Wang C.Z., Zhang Z.K. Six New Triterpenoid Saponins from the Leaves of *Ilex oblonga* and Their Inhibitory Activities against TMV Replication. Chem. Pharm. Bull. . 2007; 55(3): 422–427. https://doi.org/10.1248/cpb.55.422.

55 Zhang Z.K., Ouyang M.A., Wu Z.J., Lin Q.Y., Xie L.H. Structure–activity relationship of triterpenes and triterpenoid glycosides against tobacco mosaic virus. Planta Med. 2007; 73(14): 1457–1463. https://doi.org/10.1055/s–2007–990263.

56 Lin N., Zhang X.Q., Lei H.X., L.P. Chen, Saponin compound isolated from Rubus hirsutus as tyrosinase inhibitor for treatment of cancer, in *Faming Zhuanli Shenqing*. 2020, Lishui Hospital of Traditional Chinese Medicine, Peop. Rep. China: Peop. Rep. China

57 Lee D.Y., Yang H., Kim H.W., Sung S.H. New polyhydroxytriterpenoid derivatives from fruits of *Terminalia chebula* Retz. and their α–glucosidase and α–amylase inhibitory activity. Bioorg. Med. Chem. Lett. 2017; 27(1): 34–39. https://doi.org/10.1016/j.bmcl.2016.11.039.

58 Su X.D., Guo R.H., Yang S.Y., Kim Y.H., Y.R. Kim. Anti–bacterial effects of components from Sanguisorba officinalis L. on Vibrio vulnificus and their soluble epoxide hydrolase inhibitory activity. Nat. Prod. Res. 2019; 33(23): 3445–3449. https://doi.org/10.1080/14786419.2018.1478825.

59 Lin P., Wang Q., Liu Y., Qin Z., Gao H., M. Ye, H. Shang, X. Yao, Z. Yao. Characterization of chemical profile and quantification of representative components of DanLou tablet, a traditional Chinese medicine prescription, by UHPLC–Q/TOF–MS combined with UHPLC–TQ–MS. J. Pharm. Biomed. Anal. 2020; 180: 113070. https://doi.org/10.1016/j.jpba.2019.113070.

60 Zhou Y., Chai X.Y., Zeng K.W., Zhang J.Y., Li N., Jiang Y., Tu P.F. Ilexpublesnins C–M, eleven new triterpene saponins from the roots of *Ilex pubescens*. Planta Med. 2013; 79(1): 70–77. https://doi.org/10.1055/s–0032–1327927.

61 Li L.M., LIAO X., Peng S.L., Ding L.S. Chemical Constituents from the Seeds of *Ziziphus jujuba* var. *spinosa* (Bunge) Hu. Acta Botanica Sinica. 2005; 47: 494–498.

62 Wang Y., Liang D., Khan F.A., Zhang C.L., Y.F. Liu, R.Y. Chen, M.I. Choudhary, D.Q. Yu. Chemical constituents from *Schefflera leucantha* R.Vig. (Araliaceae). Biochem. Syst. Ecol. 2020; 91. https://doi.org/10.1016/j.bse.2020.104076.

63 He C.N., Gao W.W., Yang J.X., Bi W., Zhang X.S., Zhao Y.J. Identification of autotoxic compounds from fibrous roots of *Panax quinquefolium* L. Plant and Soil. 2008; 318(1): 63–72. https://doi.org/10.1007/s11104–008–9817–8.

64 Zeng Z., Xie R.Q., Tan L.X., Zhang T. GC–MS analysis and determination of chemical components of the extracts from Ligusticum chuanxiong Hort. by steam distillation and supercritical CO2 extraction. Chinese J. Appl. Chem. 2011; 28(8): 956–962. https://doi.org/10.3724/SP.J.1095.2011.00598.

65 Suchaichit N., Suchaichit N.P., Kanokmedhakul K., Boottanun P., Sermswan R.W., Moosophon P., Kanokmedhakul S. A new cytotoxic plumbagin derivative from roots of *Diospyros undulata*. Nat. Prod. Res. 2021; 35(10): 1605–1612. https://doi.org/10.1080/14786419.2019.1630120.

66 Kim C.S., Subedi L., Kwon O.W., Park H.B., Kim S.Y., Choi S.U., Lee K.R. Wasabisides A–E, Lignan Glycosides from the Roots of *Wasabia japonica*. J. Nat. Prod. 2016; 79(10): 2652–2657. https://doi.org/10.1021/acs.jnatprod.6b00582.

67 Puebla P., Correa S.X., Guerrero M.F., San Feliciano A. Phenylbutanoid derivatives from *Croton schiedeanus*. Biochem. Syst. Ecol. 2005; 33(8): 849–854. https://doi.org/10.1016/j.bse.2004.12.018.

68 Bonesi M., Okusa P.N., Tundis R., Loizzo M.R., Menichini F., Stévigny C., Duez P., Menichini F. Chemical Composition, Antioxidant Properties and Anti–cholinesterase Activity of *Cordia gilletii* (Boraginaceae) Leaves Essential Oil. Natural Product Communications. 2011; 6: 253–257. https://doi.org/10.1177/1934578x1100600225.

69 Wu T.Y., Cheng Y.B., Cheng F.T., Hsu Y.M., Dinh T.T., Chang F.R., Wu Y.C. Chemical Constituents of the Leaves of *Desmos cochinchinensis var. fulvescens* Ban. Helvetica Chimica Acta. 2014; 97: 1714–1718. https://doi.org/10.1002/hlca.201400108.

70 Zhu J., Shi Y., Wang H., Li M. Two new phenylpropanoid glycosides from *Lagotis brachystachya* Maxim and their Xanthione Oxidase inhibitions. Nat. Prod. Res. 2019: 1–6. https://doi.org/10.1080/14786419.2019.1662008.

71 Peng Y., Ni S.J., Li J., Li M.Y. Three new dolabrane diterpenes from the Chinese mangrove plant of *Ceriops tagal*. Phytochem. Lett. 2017; 21: 38–41. https://doi.org/10.1016/j.phytol.2017.05.018.

72 Zeng Q., Guan B., Qin J.J., Wang C.H., Cheng X.R., Ren J., Yan S.K., Jin H.Z., Zhang W.D. 2,3–Seco– and 3,4–seco–tirucallane triterpenoid derivatives from the stems of *Aphanamixis grandifolia* Blume. Phytochemistry. 2012; 80: 148–155. https://doi.org/10.1016/j.phytochem.2012.05.017.
